# Characterization of an Antennal Carboxylesterase from the Pest Moth *Spodoptera littoralis* Degrading a Host Plant Odorant

**DOI:** 10.1371/journal.pone.0015026

**Published:** 2010-11-29

**Authors:** Nicolas Durand, Gerard Carot-Sans, Thomas Chertemps, Françoise Bozzolan, Virginie Party, Michel Renou, Stéphane Debernard, Gloria Rosell, Martine Maïbèche-Coisne

**Affiliations:** 1 UMR-A 1272 UPMC-INRA Physiologie de l'Insecte, Université Pierre et Marie Curie and INRA, Paris and Versailles, France; 2 Department of Biological Chemistry and Molecular Modelling, Institute of Advanced Chemistry of Catalonia (IQAC-CSIC), Barcelona, Spain; 3 Unit of Medicinal Chemistry (associated with CSIC), Faculty of Pharmacy, University of Barcelona, Barcelona, Spain; University of California Davis, United States of America

## Abstract

**Background:**

Carboxyl/cholinesterases (CCEs) are highly diversified in insects. These enzymes have a broad range of proposed functions, in neuro/developmental processes, dietary detoxification, insecticide resistance or hormone/pheromone degradation. As few functional data are available on purified or recombinant CCEs, the physiological role of most of these enzymes is unknown. Concerning their role in olfaction, only two CCEs able to metabolize sex pheromones have been functionally characterized in insects. These enzymes are only expressed in the male antennae, and secreted into the lumen of the pheromone-sensitive sensilla. CCEs able to hydrolyze other odorants than sex pheromones, such as plant volatiles, have not been identified.

**Methodology:**

In *Spodoptera littoralis*, a major crop pest, a diversity of antennal CCEs has been previously identified. We have employed here a combination of molecular biology, biochemistry and electrophysiology approaches to functionally characterize an intracellular CCE, SlCXE10, whose predominant expression in the olfactory sensilla suggested a role in olfaction. A recombinant protein was produced using the baculovirus system and we tested its catabolic properties towards a plant volatile and the sex pheromone components.

**Conclusion:**

We showed that SlCXE10 could efficiently hydrolyze a green leaf volatile and to a lesser extent the sex pheromone components. The transcript level in male antennae was also strongly induced by exposure to this plant odorant. In antennae, SlCXE10 expression was associated with sensilla responding to the sex pheromones and to plant odours. These results suggest that a CCE-based intracellular metabolism of odorants could occur in insect antennae, in addition to the extracellular metabolism occurring within the sensillar lumen. This is the first functional characterization of an Odorant-Degrading Enzyme active towards a host plant volatile.

## Introduction

Carboxyl/cholinesterase (CCE, EC 3.1.1.1.) is a multigene family widespread in prokaryotes and eukaryotes. It includes diverse proteins that hydrolyze a broad range of carboxylic esters to their component alcohols and acids. Despite their structural and functional diversity, most of these enzymes use a same reaction mechanism based on a catalytic triad including a serine [1], [2]. Insects have multiple CCEs, as revealed by recent genome analyses [1], [3], [4], [5], [6], [7], [8], [9]: 24 genes in the bee *Apis mellifera* [3]; up to 70 in the silkmoth *Bombyx mori* [5], [6]. However, the physiological role of most insect CCEs is unknown.

Based on phylogenetic analysis and substrate specificities, insect CCE genes have been classified into 33 major clades [10] and three major classes [3]. The first class contains proteins implicated in neuro/developmental functions [11]. This group includes acetylcholinesterases, neuroligins [11], gliotactins and other uncharacterized proteins [1]. Except acetylcholinesterases, proteins of this class are generally considered to be non-catalytic, such as neuroligins involved in cell-cell interactions in synapses [11].

The second class contains mostly secreted and generally catalytically active enzymes. For a few, functional data suggest their involvement in hormone or odorant processing. Juvenile hormone (JH) esterase (JHE) is involved in the degradation of JH, a key hormone regulating development, metamorphosis and reproduction in insects [12] and is one of the few insect esterases other than acetylcholinesterases to have a clearly defined substrate. CCEs potentially involved in sex pheromone degradation have been identified in several insect species, especially in moths [13], [14], [15], [16], [17], [18], [19]. These Pheromone Degrading Enzymes (PDEs) are generally specifically expressed in the antennae, the insect olfactory organs. Antennae carry hair-like structures called olfactory sensilla, which enclose olfactory receptor neurons (ORNs) embedded by accessory cells and surrounded by a protein-enriched lymph [20]. Extracellular PDEs are supposed to degrade the pheromone components within the lymph, in the vicinity of the olfactory receptors located in the ORN membrane [13]. Rapid degradation of female sex pheromone in male antennae is believed to play an essential role during male flight towards pheromone trail [18]. However, ability of PDEs belonging to the CCE family to hydrolyze sex pheromone components has been demonstrated in only two species, the moth *Antheraea polyphemus* and the beetle *Popillia japonica* [17], [18], [19]. Moreover, CCEs able to hydrolyze other odorants than sex pheromones have not been characterized yet.

The third class contains predominantly intracellular active enzymes. Few functional data are available on these enzymes. Most of them have been proposed to have digestive or detoxification function, based on their expression in insect midgut, or have been implicated in insecticide resistance [1], [10]. CCE-related insecticide resistance has been intensely studied in insects and two mechanisms have been demonstrated, i.e. mutation in amino acid sequences and gene overexpression [21], [22]. The role of CCEs in the adaptation of insects to ingested plant chemicals has not been so greatly documented (reviewed in [23]).

In *Spodoptera littoralis*, a worldwide pest of cotton and vegetable crops, analysis of an Expressed Sequence Tag collection from male antennae has revealed a high diversity of CCEs expressed in the olfactory organs [24]. Among them, a gene belonging to the third CCE class and encoding a putative intracellular esterase (*SlCXE10*, GenBank accession number FJ652453) was of particular interest, as preliminary RT-PCR experiment revealed that it was strongly expressed in antennae of both sexes. As the involvement of this class of CCEs in olfaction was unknown, we investigated if *SlCXE10* could have a role in odorant hydrolysis. In our study, we characterized the tissue specific and developmental expression patterns of *SlCXE10*, and we expressed recombinant SlCXE10 protein and tested its activity *in vitro* towards physiological relevant odorants. For our functional studies, we focused on the pheromone components (*Z*,*E*)-9,11-tetradecadienyl acetate (*Z*9*E*11-14:Ac) and (*Z*,*E*)-9,12-tetradecadienyl acetate (*Z*9*E*12-14:Ac) and the plant volatile (*Z*)-3-hexenyl acetate (*Z*3-6:Ac). The sex pheromone of female *S. littoralis* is a mix of two esters, *Z*9*E*11-14:Ac and *Z*9*E*12-14:Ac. Responses of males to these compounds have been well characterized at the electrophysiological [25] and behavioural levels [26] and females were also able, to some degree, to detect them [25]. *S. littoralis* also responds to the host plant volatile *Z*3-6:Ac. The ability to detect green leaf volatiles was mostly described in females [27], [28], because these components play a crucial role for host plant selection before egg laying. Few esters from *S. littoralis* host plants have been identified and tested in electrophysiology. Among those which have been tested, *Z*3-6:Ac induced clear antennal responses in *S. littoralis* females; male responses were not studied. We thus confirmed that males are electrophysiologically responsive to Z3-6AC to validate its use as a potential substrate for recombinant SlCXE10.

Our results demonstrated for the first time, that an insect intracellular CCE, predominantly expressed in antennae of both sexes and associated with olfactory sensilla, was able to hydrolyze a plant volatile and to a lesser degree the sex pheromone components. We also showed that the transcript level in males was up regulated by the exposition to this plant odorant. These results open a new range of potential substrates for CCEs belonging to the class 3, in addition to insecticides and dietary plant chemicals.

## Results

### Tissue-related and temporal expression of *SlCXE10*

**Figure 1:**
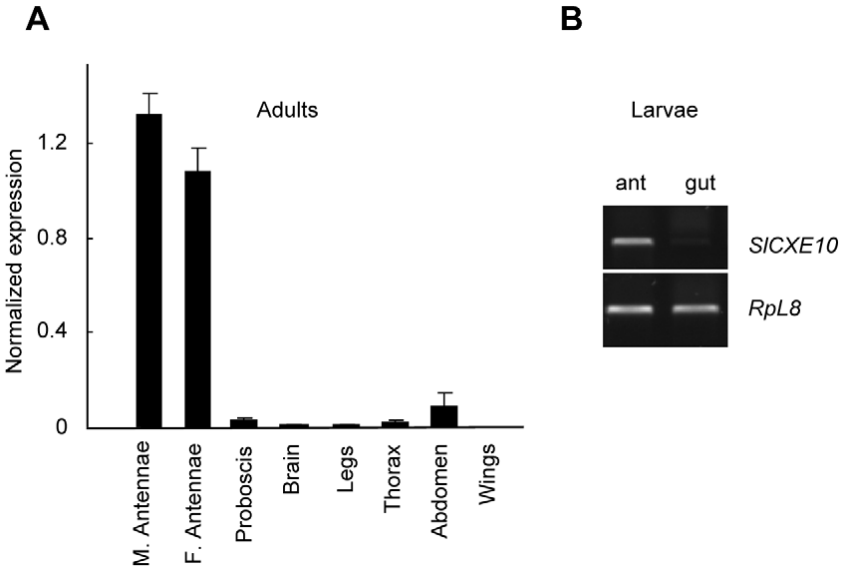
Analysis of *SlCXE10* expression in *S. littoralis* tissues. **A**) Quantitative PCR (qPCR) analysis on cDNAs from male adult tissues and from female adult antennae. The expression level of *SlCXE10* was normalized to that of *RpL13* transcript, which was measured in the same cDNAs. Data were obtained from triplicate experiments and are given as the mean ±SD. M: male; F: female. **B**) RT-PCR analysis on cDNAs from last instar larval antennae and midguts.

We determined the tissue distribution and expression levels of the *SlCXE10* transcripts in adults by a quantitative PCR (qPCR) method. The expression levels of *SlCXE10* in various tissues were shown as relative amounts compared with the *RpL13* transcripts (Fig. 1A). The *SlCXE10* transcripts were highly expressed in the antennae of both sexes, with a slightly higher level in males than in female antennae (1.2-fold). *SlCXE10* expression was at least 15-fold higher in antennae than in non-olfactory tissues. *SlCXE10* was expressed in abdomen at a relatively low level. The expression in other tissues was barely detectable. In last instar larvae, expression was clearly detected in antennae but not in midguts, as revealed by RT-PCR analysis (Fig. 1B).

**Figure 2:**
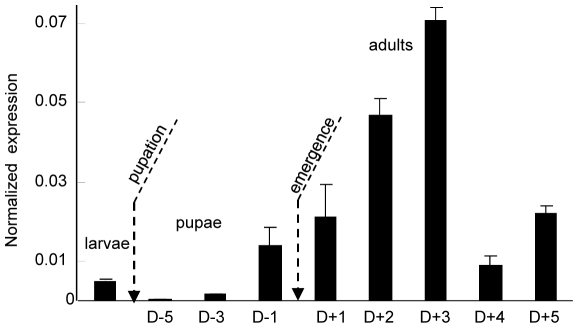
qPCR analysis of *SlCXE10* expression in male antennae during development and adult life. The expression level of *SlCXE10* was normalized to that of *RpL13*. Data were obtained from triplicate experiments and are given as the mean ±SD.

In antennae, the expression of *SlCXE10* during male post-embryonic development and adult life was studied more precisely by qPCR (Fig. 2). *SlCXE10* expression was again detected in the antennae of last instar larvae then its level decreased at the beginning of pupal development. RNA levels increased steadily from the end of the pupal stage until adult emergence, reaching a maximum three days after emergence and then decreasing abruptly.

### *SlCXE10* localization within antennae

**Figure 3:**
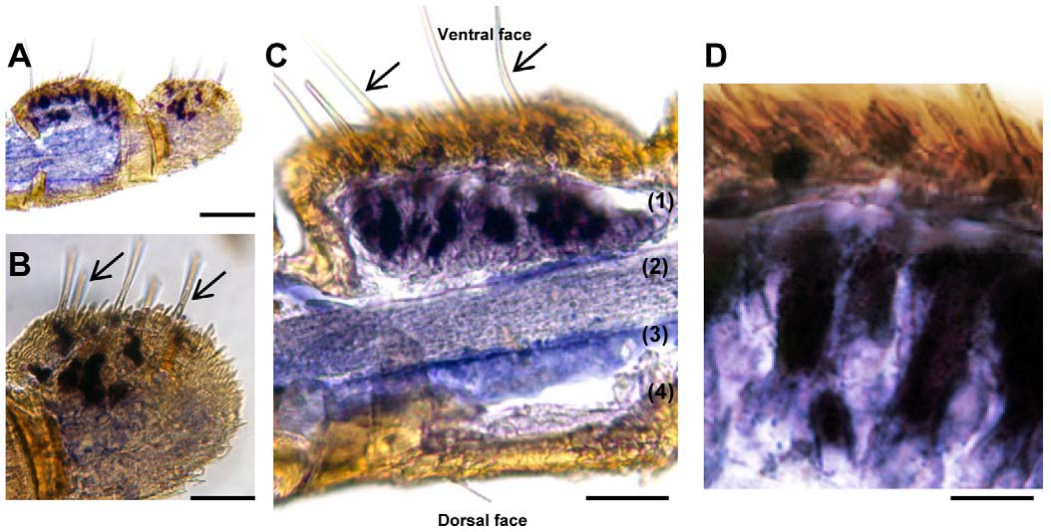
Expression patterns of *SlCXE10* after *in situ* hybridization on longitudinal sections of *S. littoralis* male antennae. Arrows show the olfactory sensilla. **A**) Global view of two antennal segments, one sectioned and the other one with intact cuticle. **B**) Higher magnification of A, showing labeling at the base of the olfactory sensilla. **C**) Longitudinal section through a segment showing the disposition of the olfactory epithelium (1), the antennal nerve (2), the antennal lumen filled with haemolymph (3) and the epidermis (4). **D**) Higher magnification of C showing labeled cells (accessory cells and/or neurons). Scale: 100 µm in A; 50 µm in B and C, 20 µm in D.

The cellular localization of *SlCXE10* transcripts in male antennae was characterized by *in situ* hybridization (Fig. 3). *S. littoralis* male antennae are filiform, with olfactory sensilla on the ventral side and only scales on the dorsal side [25]. Male antennae have both long and short trichoid sensilla: the longest ones are distributed in the medial and lateral ventral region while the shortest are predominantly in the medial ventral region. Some short basiconic sensilla are also located in the medial ventral region [29]. After *in situ* hybridization, *SlCXE10* signals were restricted to the sensilla side of the antennae, with no labelling on the scale side (Fig. 3, A, C). Labelling was located in cells at the base of the olfactory sensilla (Fig. 3, C, D). On longitudinal sections, the distinction between long and short sensilla was not possible. However, in some antennal segment with intact cuticle (Fig. 3, B), the labelling was observed all over the ventral and lateral surface suggesting an expression of *SlCXE10* in both long and short olfactory sensilla. Sense probes gave no signals.

### Production and purification of SlCXE10 recombinant protein

**Figure 4:**
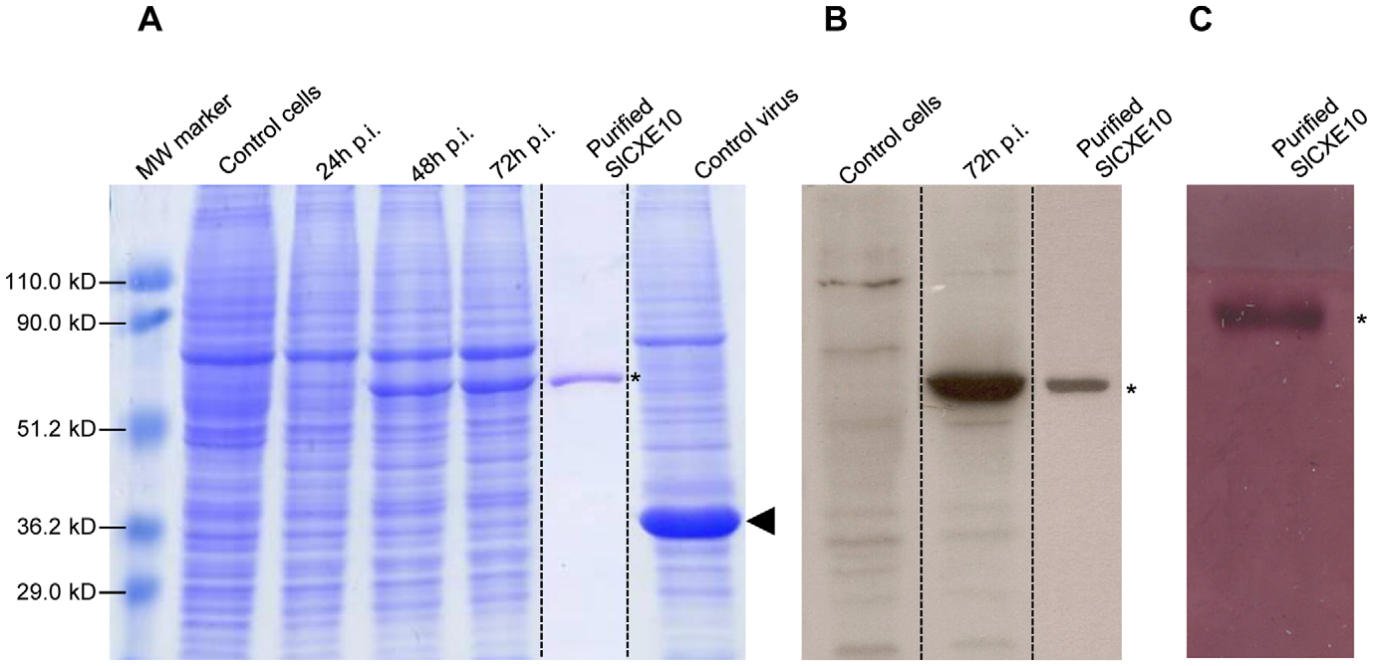
SlCXE10 recombinant protein expression and purification. **A**) SDS PAGE analysis of total proteins extracted from infected insect cells at different times after infection (24, 48, 72 h p.i). Non-infected cells and cells infected by a non-recombinant virus were used as controls. **B**) Western-blot analysis. **C**) α/β-naphthyl acetate staining. SlCXE10 are indicated by asterisks on the right side of the gel, polyhedrin is indicated with an arrowhead. The sizes of the molecular-mass markers are shown on the left hand side of the gel.

Recombinant SlCXE10 was produced using a baculovirus expression system in lepidopteran *Sf*21 cells. The expression of the recombinant protein was analyzed by SDS PAGE of infected insect cells at different times after viral infection (Fig. 4A). Expression of the recombinant protein occurred after 48 h post-infection (p.i.), corresponding to the time of activation of the “very late polyhedrin promoter” which was driving expression. Non-infected cells were used as negative control as well as cells infected with a non recombinant virus, which produced polyhedrin normally. Recombinant SlCXE10 had a molecular mass of about 60 kDa, consistent with the predicted molecular mass of 61 kDa based on translation of the complete ORF [24]. Using western-blot analysis, a single band with the same molecular mass was observed before and after the purification steps (Fig. 4B). A single band was also detected by Coomassie staining following purification. Native PAGE followed by an α/β-naphthyl acetate assay on purified recombinant SlCXE10 confirmed that the enzyme was still active following purification (Fig. 4C).

### Antennal response to *Z*3-6:Ac

**Figure 5:**
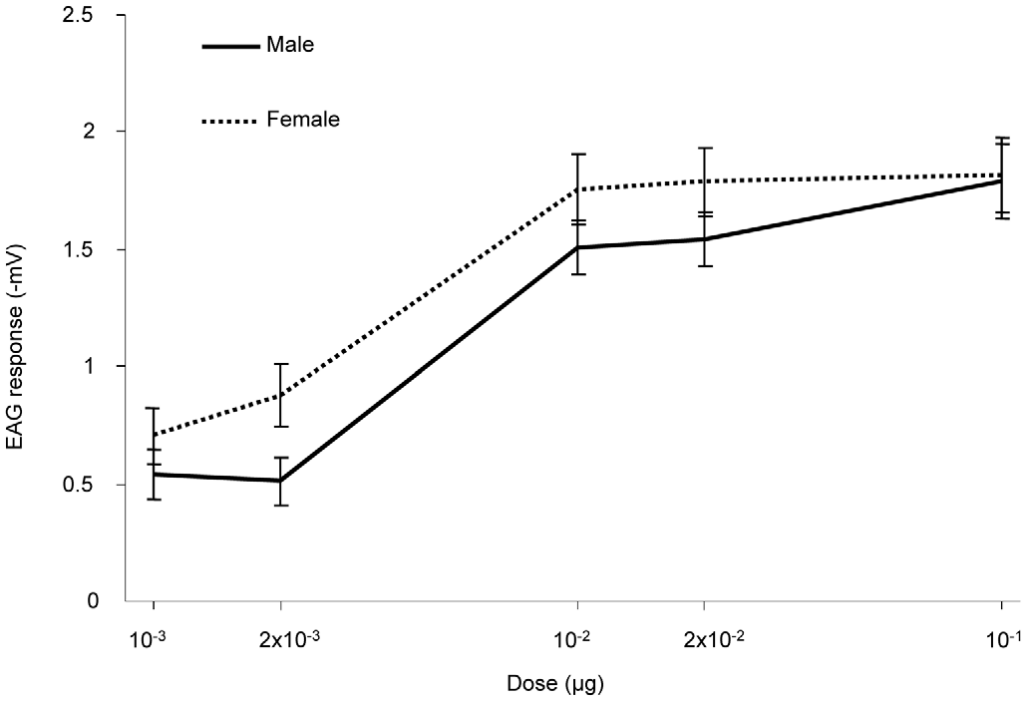
Electroantennographic responses to *Z*3-6:Ac. Full line and dotted line represent male and female responses, respectively (mean±SEM, *n* = 17 and 12, respectively).

To test if males were responsive to *Z*3-6:Ac, we performed an electrophysiological study. Responses were quantified by measuring the amplitude of antennal depolarization after odorant stimulation. Antennae of both sexes responded to *Z*3-6:Ac in a dose-dependent manner (Fig. 5). Male responses were slightly weaker than the female ones for odorant doses ranging from 10^−3^ to 2×10^−2^ µg. At higher doses (10^−1^ µg), the responses were comparable in both sexes. When using mineral oil alone, antennal responses were of −0.03 mV in males and 0 mV in females.

### Kinetic study

**Figure 6:**
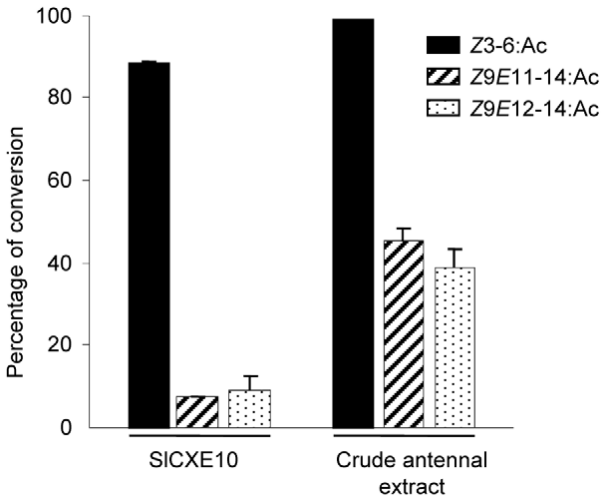
Hydrolysis of Z3-6:Ac, Z9E11-14:Ac and Z9E12-14:Ac by recombinant SlCXE10 and crude antennal extracts. Hydrolysis was indicated by the percentage of conversion of the esters in the corresponding alcohols after 1 h of incubation. Data were obtained from triplicate experiments and are given as the mean ±SD.

**Figure 7:**
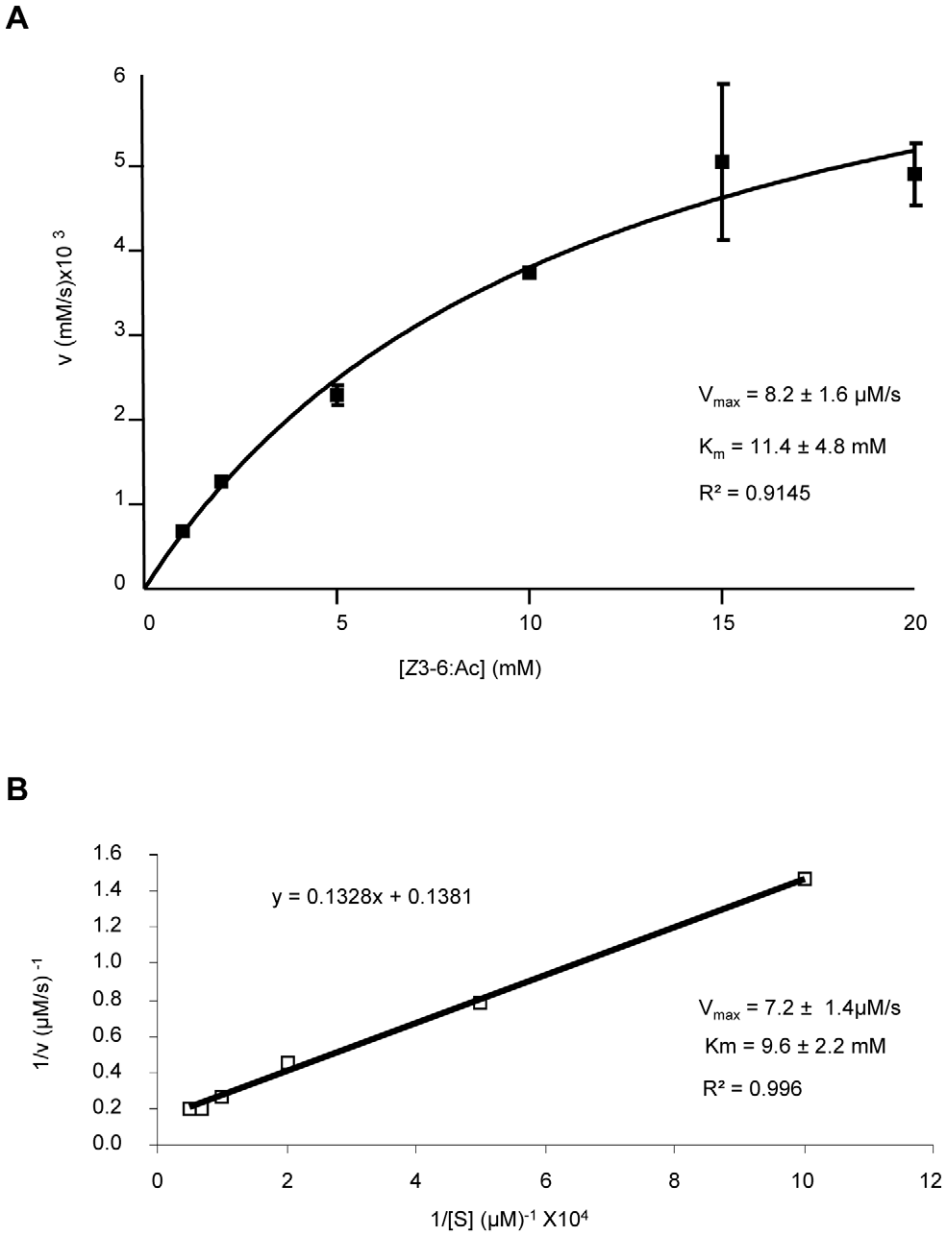
Kinetics of *Z*3-6:Ac hydrolysis by SlCXE10. **A**) Nonlinear regression analysis. **B**) Lineweaver-Burk representation.

The ability of recombinant SlCXE10 to degrade the sex pheromone components (*Z*9*E*11-14:Ac and *Z*9*E*12-14:Ac) and the plant volatile *Z*3-6:Ac was analyzed by GC and GC-MS; crude antennal extracts were used as positive controls. Hydrolysis was indicated by the percentage of conversion of the acetates to the corresponding alcohols after 1 h of incubation. For the crude antennal extract, hydrolysis of *Z*9*E*11-14:Ac and *Z*9*E*12-14:Ac was around 40%, as previously reported [30], while hydrolysis of *Z*3-6:Ac was nearly 100% (Fig. 6). For recombinant SlCXE10, hydrolysis of *Z*9*E*11-14:Ac and *Z*9*E*12-14:Ac was 7.63% and 8.98% respectively, while hydrolysis of *Z*3-6:Ac was 89.2%. The high level of activity allowed the calculation of kinetic values for recombinant SlCXE10 and *Z*3-6:Ac; a K_m_ of 11.4±4.8 mM and a V_max_ of 8.2±1.6 µM/s was determined by non-linear fitting (R^2^ = 0.9145) and a K_m_ of 9.6±2.26 mM and a V_max_ 7.2±1.48 µM/s, respectively, using the Lineweaver-Burk method (R^2^ = 0.996), (Fig. 7).

### Effect of odorant exposure on *SlCXE10* expression level in males

**Figure 8:**
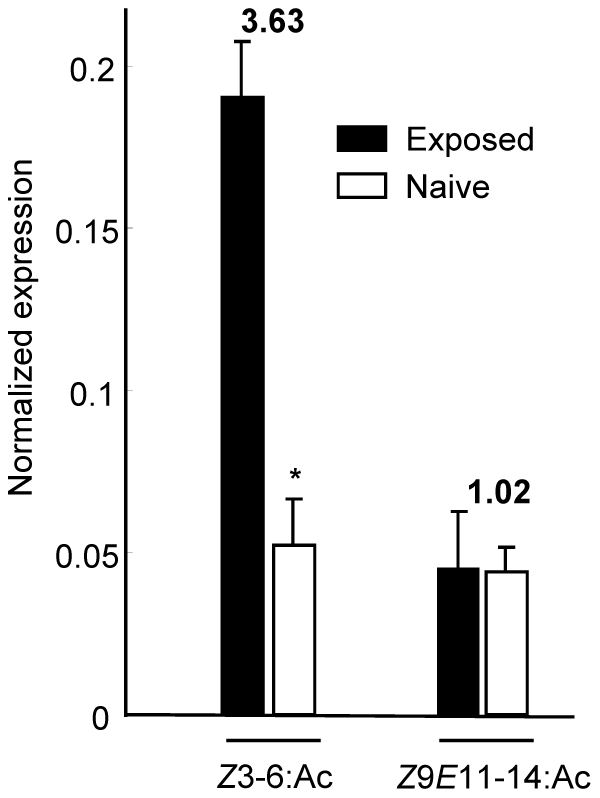
Quantitative analysis of SlCXE10 transcripts in male antennae after *Z*3-6:Ac and *Z*9*E*11-14:Ac exposition by qPCR. The expression level of *SlCXE10* was normalized to that of *RpL13*. Data were obtained from triplicate experiments and are given as the mean ±SD.

To test the hypothesis that *SlCXE10* expression could be induced by exposing animals to odorants, males were exposed for 48 h to high doses of *Z*3-6:Ac (plant volatile) and *Z*9*E*11-14:Ac (pheromone component). Levels of *SlCXE10* transcripts were compared between naive and exposed males by quantitative PCR (Fig. 8). Male antennae exposed to *Z*3-6:Ac expressed 3.63 times more *SlCXE10* than the controls (p = 0.0021, Student t-test). The transcript levels were comparable when males were exposed to *Z*9*E*11-14:Ac.

## Discussion

*SlCXE10* was strongly and predominantly expressed in the adult antennae compared to all other tissues tested. This expression pattern suggested that SlCXE10 could be implicated in a specific olfactory function. Indeed, such restricted expression is a useful criterion that has already been helpful in identifying specific olfactory genes such as olfactory receptors and Odorant-Binding Proteins (OBPs) in various species [31].

During development, *SlCXE10* expression level increased from the end of the pupal stage and reached a maximum in 3-day-old adults. Apol-PDE, a CCE clearly involved in sex pheromone degradation in the moth *Antheraea polyphemus* presented a similar profile [18]. These expression patterns were concomitant with the electrophysiological responsiveness to odorant components and with the expression of other olfactory genes such as OBPs [18], [32], [33] or ODEs [34], suggesting a common regulatory pathway for the genes involved in odorant detection in mature adult moths. The life of *S. littoralis* males is short: they are able to mate rapidly after emergence; they survive as adults for 5 to 6 days in our rearing conditions. Maximum levels of *SlCXE10* expression in males were thus consistent with their short adult lifespan.

Interestingly, *SlCXE10* transcripts were also clearly detected in antennae from last instar larvae but not from the larval midgut. This is the first observation of expression of a presumptive ODE in insect larval antennae. The majority of CCEs identified during this stage were indeed generally associated with the digestive tract [6], [10], suggesting a role in detoxification of noxious substances in larval diet. In many insect larvae, olfactory stimuli play a role for food location and studies in *Drosophila melanogaster* showed that the organization and functioning of the olfactory system from larvae is simpler than but similar to the adult system, from the odorant reception to the odorant-driven behavior (reviewed in [35]). Antennal larval CCEs could thus play a role in odorant degradation, as assumed in adults.

In the adult male antennae, *SlCXE10* expression was restricted to the olfactory sensilla, as revealed by *in situ* hybridization. Expression was associated with long and short olfactory sensilla, in cells that could correspond to accessory cells and/or to the olfactory neurons. In male *S. littoralis*, most of the olfactory sensilla are long trichoid sensilla narrowly specialized in *Z*9*E*11-14:Ac and *Z*9*E*12-14:Ac detection [25]. Short olfactory sensilla (trichoid and basiconic) are tuned to various plant odours [28] but their responses to plant esters were not tested in males. On the contrary, most of the sensilla from female antennae are short and specialized in the detection of plant odours, including *Z*3-6:Ac [27], [28]. Our electrophysiological study demonstrates that males can also detect *Z*3-6:Ac presumably via the short sensilla. In male antennae, *SlCXE10* expression was thus observed to associate with olfactory sensilla which respond to either sex pheromone or host-plant esters. Therefore, we hypothesize that SlCXE10 has a role in the hydrolysis of both pheromone and plant volatile compounds.

In our *in vitro* conditions, recombinant SlCXE10 was indeed able to degrade these three esters but with a strong preference to *Z*3-6:Ac compared to *Z*9*E*11-14:Ac and *Z*9*E*12-14:Ac. The differences in terms of carbon chain length, unsaturations and hydrophobicity between *Z*3-6:Ac and sex pheromone compounds could easily account for this difference. Towards *Z*3-6:Ac, recombinant SlCXE10 is fast, with a *in vitro* turnover number of 45 s^−1^. This turnover number is higher than of insect JHE (0.6–4.3 s^−1^, reviewed in [1]). It ranged between the k_cat_ values of the two CCEs previously characterized as PDEs in *A. polyphemus* (Apol-PDE) and in *P. japonica* (Pjap-PDE): k_cat_ values for the sex pheromone of 127 s^−1^ [18] and 1.36 s^−1^ (calculated from [17]), respectively. SlCXE10 affinity for *Z*3-6:Ac is quite low, leading to a moderate specific activity (K_m_/k_cat_) of 4.7×10^3^ M^−1^ s^−1^. This activity is smaller than that of ApolPDE (1×10^8^ M^−1^ s^−1^), of insect JHEs (6–501×10^6^ M^−1^ s^−1^, [1]) or of a malathion-CCE from *Lucilia cuprina* (2.7×10^6^ M^−1^ s^−1^) [36] but is higher than that of Pjap-PDE (2×10^3^ M^−1^ s^−1^, calculated from [17]).

This high K_m_ towards *Z*3-6:Ac revealed that the affinity of SlCXE10 for Z3-6:Ac is low and suggested that other exogenous or endogenous compounds might be additional substrates for the native enzyme. *SlCXE10* expression level in adult male antennae was nevertheless clearly induced by Z3-6:Ac exposition *in vivo* and not by the sex pheromone component *Z*9*E*11-14:Ac. As many studies showed that the enzyme substrates are capable of inducing the expression of those enzymes, this suggested that *Z*3-6:Ac might be a physiological substrate for SlCXE10 in adult males. Induction of detoxification enzymes, including CCEs, by xenobiotics or plant allelochemicals present in the diet has been well studied in insects (reviewed in [23], [37]). We demonstrate here for the first time that an odorant molecule could also be an effective CCE inducer in insects. Enzyme over-expression may increase odorant clearance and thus avoid adaptation/habituation to the odorant signals. This mechanism could participate to maintaining the specificity of olfactory communication in a noisy environment, e.g. when males have to detect minute amounts of sex pheromone in a high background of various plant volatiles.

SlCXE10 lacks a secretory signal peptide as well as the C-terminal endoplasmic retrieval signal [1], suggesting a localization into the cytosol of the accessory cells (and/or ORNs) of the sensilla. In contrast to the extracellular PDEs present in the sensillar lymph, SlCXE10 can thus presumably act on Z3-6:Ac and putatively other odorants only after their entry to the cells. It has been suggested that the complexes OBP-odorant and OR-odorant are internalized into the support cells and ORNs, respectively [38], [39]. Because of their hydrophobicity, the odorant molecules tested here may also directly enter the cells across their plasma membranes. Our results strongly suggest that a CCE-based intracellular metabolism of odorants could occur in insect antennae, in addition to the extracellular metabolism already proposed. Antennal intracellular cytochromes P450 and glutathione S-tranferases involved in pheromone or odorant degradation have been described [40], [41]. This complex metabolism could participate in the clearance of odours within the antennae.

Further studies will allow to precise the catalytic properties of SlCXE10 to establish if this enzyme may also detoxify xenobiotics entering the sensilla, as suggested for intracellular CCEs expressed in larval midgut.

## Materials and Methods

### Chemicals

*Z*9*E*11-14:Ac and *Z*9*E*12-14:Ac were synthesized in the laboratory (courtesy of Martine Lettere, >97% purity checked by gas chromatography, CAS 50767-79-8 and 30507-70-1, respectively). *Z*3-6:Ac was purchased from Lancaster Synthesis (Alpha Aesar, USA; 99% purity, CAS 3681-71-8). (*Z*)-3-hexenol (*Z*3-6:OH, 99% purity, CAS 928-96-1) and 5-methyl-1-hexanol (99% purity, CAS 627-98-5) were purchased from Sigma-Aldrich. Substrates were diluted in hexane (>98% purity, CAS 110-54-3, Carlo-Erba).

### Animals and tissue collection

Insects were reared on semi-artificial diet at 24°C, 60–70% relative humidity, and under a 16∶8 h light:dark (LD) photoperiod till emergence. Sexes were separated at pupal stage. Adults were kept under an inverted LD regime and provided with a 10% sucrose solution. For *SlCXE10* expression analysis by polymerase chain reaction (PCR), various tissues were dissected: antennae and midguts from last instar feeding larvae, antennae from male pupae and from adults, male proboscis, brains, legs, thorax, abdomens and wings. For odorant exposure experiments, 15 one-day-old males were set during 48 h into hermetically sealed boxes containing either 1 µg of *Z*3-6:Ac or *Z*9*E*11-14:Ac loaded onto a filter paper. Insects were also provided with a 10% sucrose solution. Control animals were kept in the same conditions except that the filter paper was only loaded with hexane. Antennae were then dissected. For *in situ* hybridization, male antennae were fixed overnight at 4°C in 4% PFA-3% Tween 20, rinsed in PBS (0.85% NaCl, 1.4 mM KH_2_PO_4_, 8 mM Na_2_HPO_4_, pH 7.1; Sigma) and then cryoprotected in PBS-18% sucrose at 4°C before use. For kinetic studies, two-day-old male antennae were dissected and immediately used for crude antennal extracts preparation.

### RNA isolation and cDNA synthesis

Total RNAs were extracted with TRIzol Reagent (Invitrogen, Carlsbad, CA, USA), then treated with DNase I (Roche, Basel, Switzerland) and quantified with a spectrophotometer (BioPhotometer, Eppendorf, Hamburg, Germany). Single-stranded cDNAs were synthesized from total RNAs (5 µg) from various tissues using Superscript II reverse transcriptase (Gibco BRL, Invitrogen) with the oligo(dT)_18_ primer according to the manufacturer's instructions.

### Expression analysis of *SlCXE10* by quantitative RT-PCR (qPCR) and RT-PCR

Two specific primers, SlCXE10-q.F (5′-CGGACGACCGGTCAGTTGTA) and SlCXE10-q.R (5′-TACCAGGGACCAGCGTGTTG) were designed using EPRIMER3 software (http://mobyle.pasteur.fr/cgi-bin/MobylePortal/portal.py?form=eprimer3). The reference genes (*RpL13*, *RpL8*, *GAPDH and β-actin*) and their corresponding primers were described in [24]. All reactions were performed on the LightCycler® 480 Real-Time PCR System (Roche, Basel, Switzerland). Each 16 µl reaction consisted of 8 µl LightCycler® 480 SYBR Green I Master (Roche, Basel, Switzerland), 6 µl of 12-fold diluted cDNA (or water for negative control) and 1 µl of each primer. The qPCR program consisted of 95°C for 13.5 min, then 40 cycles of 95°C for 30 s, 60°C for 30 s, 72°C for 30 s. This was followed by the measurement of fluorescence during a 55 to 95°C melting curve in order to detect a single gene-specific peak and to check the absence of primer dimer peaks. A negative control and a fivefold dilution series of pooled cDNAs (from all conditions) were included in each run. The fivefold dilution series were used to construct a relative standard curve to determine the PCR efficiencies and for further quantification analysis. Each reaction was run in triplicate (technical replicate) with at least three independent biological replicates. Data were analysed with LightCycler 480® Software (Roche, Basel, Switzerland) and the crossing point values (Cp-values) were first determined for the reference genes with a run formed by the fivefold dilution series, the measuring points and three negative controls. The *RpL13* gene was considered as displaying consistent expression and was suitable for downstream analysis, as previously described in [24]. Subsequently, the expression of *SlCXE10* was normalized to geometric means of this reference and the normalized gene expression was then calculated with Q-Gene software [42].

Non-quantitative RT-PCR was performed on 100 ng of cDNAs from larval antennae and midguts, using SlCXE10-q.F/q.R and *RpL8* primers. 30 cycles of amplification were realized for *SlCXE10* and 25 for *RpL8* in order to fit the linear range of amplification.

### *In situ* hybridization

A cDNA fragment of 601 bp was amplified by PCR using SlCXE10-ish.F (5′-AGCTATTTAGGTGAACACTATAGTGAATTCTTCAAAAAACAACCTTGTG) and SlCXE10-ish.R (5′-ATTGTAATACGACTCACTATAGGGTCTCCGTAAGATGGCC) primers and was used as template for *in vitro* transcription to generate DIG-labeled RNA sense and antisense probes. Antennae from 2-day-old male moths were embedded in Tissue-Tek medium™ compound (CellPath, Newtown Powys, UK). Cryosections (7 µm) were set in cell culture insert (Greiner Bio-one, Monroe, USA) and rinsed twice in PBS for 5 min and twice in 2xSSC for 5 min. Sections were first incubated for 1 h at 45°C in hybridization solution (50% formamide, 5x SSC, 5x Denhart's solution, 50 µg/µl yeast tRNA, 4 mM EDTA, 2.5% Dextran), then hybridization was conducted overnight at 45°C in the same solution containing the labeled probe. Post-hybridization, sections were washed 10 min in 2xSSC, incubated 30 min at 37°C with RNase, rinsed twice in 0.1xSSC for 30 min at 65°C and 5 min in PBS. After blocking with 0.5% blocking reagent (Roche) for 30 min at RT, sections were incubated with anti-Dig AP conjugated antibody diluted 1∶500 for 30 min at RT. After 5 min washing in detection buffer (100 mM Tris pH 9.5, 100 mM NaCl, 50 mM MgCl_2_, 0.1% tween 20, 20 mM levamisole), hybridized probes were visualized by incubation in the dark with NBT-BCIP as substrates in detection buffer. Reaction was stopped by rinsing sections in detection buffer. Sections were mounted on slides and pictures were acquired (Olympus BX61 microscope, ImagePro software) and digitalized using Adobe Photoshop® 7.0 (Adobe, USA).

### Construction of recombinant baculovirus SlCXE10-AcMNPV

The *SlCXE10* coding region was amplified from male antennal cDNA by PCR using Expand High Fidelity PCR system (Roche, Bazel, Switzerland) and two specific primers (SlCXE10-ORF.F: 5′-GCGATGGTGCAAGTGAGAGTGAGCGAGGGTGTA and SlCXE10-ORF.R: 5′-TTGATTTAGCGTACTAAATTTAGGCAGGTG). The 1.63 kb PCR product was cloned into pBlueBac4.5/V5-His TOPO transfer plasmid (Invitrogen). The presence of the insert, in-frame with the polyhedrin promoter from *Autographa californica* multi nuclear polyhedrosis virus (AcMNPV), as well as the presence of the carboxy-terminal hexahistidine tag (6His-tag), were confirmed by sequence analysis (GATC Biotech, Marseille, France). This plasmid DNA was cotransfected with viral DNA into *Spodoptera frugiperda Sf*21 cells using the bacmid DNA-CellFECTIN mixture (Bac’N’Blue, Invitrogen). Recombinant viruses were isolated from the transfection supernatant by plaque purification. Occlusion-positive plaques, representing recombinant viruses, were picked and plaque purified. Single isolated recombinant viruses were amplified to obtain high-titre virus stocks. Virus titres were determined by plaque assays. A concentrated viral stock (1×10^7^ pfu/ml) was stored at 4°C for further experiments.

### Expression and purification of recombinant SlCXE10

*Sf*21 cells (5×10^5^ cells/ml) in 5 ml of Sf-900 II (Invitrogen) were infected with the viral stock at a multiplicity of infection of 20 and grown at 27°C for 72 h. Cells were washed twice with PBS, then cell pellet was diluted with a lysis buffer (1% Nonidet P40, 50 mM NaH_2_PO_4_, 300 mM NaCl, 10 mM imidazole, pH 8) and incubated on ice for 10 min. Cells debris were removed by centrifugation, the supernatant was isolated and 200 µl of nickel-charged resin (Ni-NTA Agarose, Qiagen) was added before 1 h incubation at 4°C under agitation. Suspension was loaded into a polypropylene column, washed twice with wash buffer (50 mM NaH_2_PO_4_, 300 mM NaCl, 20 mM imidazole, pH 8) and eluted with elution buffer (50 mM NaH_2_PO_4_, 300 mM NaCl, 250 mM imidazole, pH 8). Protein purification was controlled by polyacrylamide gel electrophoresis (PAGE) and Western-blot analyses. Elution fractions were separated by SDS PAGE and either visualized by Coomassie staining or transferred to a nitrocellulose membrane for western-blot: after blocking in TBST-10% blocking reagent (Invitrogen), membranes were incubated overnight at 4°C with anti-6His-tag primary antibody (Sigma-Aldrich, 1∶10,000), then incubated with horseradish-peroxydase-labelled antibody (Sigma-Aldrich, 1∶10,000). Blots were washed and incubated with chemoluminescent substrate for 1 min (ECL Plus Western Detection Kit, GE Healthcare). To check enzyme activity, elution fractions were subjected to native PAGE and esterase activities were visualized by α/β-naphthyl acetate assay, as described in [16].

### Electrophysiology

Two-day-old male and female moths were anesthetized with CO_2_ and restrained in a styrofoam holder. A chlorinated silver wire was inserted into the abdomen to serve as reference electrode. One antenna was fixed with small strips of adhesive tape on the surface of the holder and the tip of the antenna was cut off. The recording electrode, filled with sensillum saline (10^−3^ M Ca^2+^ solution after [43] modified from [44]) was slipped over the end of the cut antenna. Both electrodes were connected to a preamplifier NL 102 (Digitimer, Hertfordshire, UK). The signal was amplified (x 1000) and filtered from DC to 10 kHz and digitized at 10 kHz and 12 bits with a Data Translation DT3001 board (Data Translation, Malboro, USA). The amplitude of electroantennograms was measured using Awave software [45]. Olfactory stimuli were delivered with a programmable olfactometer that used distinct sources of Z3-6:Ac for different dilutions as described in [46]. White mineral oil from Sigma (CAS 8042-47-5) (Sigma-Aldrich, L’Isle-d’Abeau, France) was used to prepare 1/1000, 1/500, 1/100, 1/50, 1/10 volume to volume dilutions. The antenna was permanently bathed by pure air at 440 ml/min. During stimulation periods, pure air was replaced by *Z*3-6:Ac or control (mineral oil). Every antenna was challenged with a series of six stimulations: mineral oil, 1/1000, 1/500, 1/100, 1/50 and 1/10 *Z*3-6:Ac solution. Odorant or control was applied as a short pulse of 500 ms. Experiments were repeated on 17 males and 12 females.

### Kinetic study

Crude antennal extracts were prepared by homogenization of antennae on liquid nitrogen in 20 mM Tris-HCl buffer at pH 7.4. Homogenates were briefly sonicated, centrifuged at 12000 rpm for 5 min and the supernatants were stored at 4°C until rapid use. To study the kinetics of odorant hydrolysis by antennal extracts and recombinant SlCXE10, the production of the corresponding alcohols was monitored. 500 ng of freshly purified recombinant protein or 4 µg of male antennal extract were incubated during 1 h at 28°C in 50 µl of 20 mM Tris buffer (pH 7.4) with either *Z*3-6:Ac, *Z*9*E*11-14:Ac or *Z*9*E*12-14:Ac (final concentration: 40 µM). No detergent was used. Substrate and product were extracted immediately with a mix of ether and hexane (1∶1 v/v). The organic phase was separated and concentrated before injection of the totality of the sample in gas chromatography (GC, Thermo Finnigan Trace GC; HP-5 Agilent column). Identification of the product was confirmed by mass spectrometry analysis (Thermo Finnigan Trace GC-MS). The GC conditions for the sex pheromone components were as follows: injection at 80°C, hold for 1 min, 5°C/min up to 220°C, 10°C/min up to 300°C and 5 min of hold at this temperature. For Z3-6:Ac, injection was performed at 50°C, hold for 1 min, followed by 1°C/min up to 65°C, 5°C/min up to 80°C, 10°C/min up to 300°C and 5 min of hold at this temperature. Three replicates for each substrate were analyzed. The percentage of conversion was calculated by the relative amount of the derived alcohol with regard to the parent acetate. For K_m_ and V_max_ determination, 2 µg of purified recombinant SlCXE10 were incubated in a 200 µl reaction mixture with various concentrations of *Z*3-6:Ac (1 mM to 20 mM). After 5 min of incubation, substrate and product were extracted immediately with 400 µl of ether/hexane mix (1∶1 v/v) containing 5-methyl-1-hexanol as internal standard. The GC detector was calibrated with a wide range of concentrations of *Z*3-6:OH and standard diluted in hexane. Experiments were replicated twice for each concentration. Kinetic parameters were determined by fitting the experimental activity data to the one site binding equation of GraphPad Prism 5 as well as by Lineweaver-Burk plots (double reciprocal, 1/*v* versus 1/[S]).
